# Uncovering hidden patterns: use of infant feeding profiles in the first 6 months postpartum to inform the effectiveness of breastfeeding promotion programs

**DOI:** 10.3389/fpubh.2026.1754431

**Published:** 2026-04-17

**Authors:** Siva Balakrishnan, Yunwei Chen, Gary L. Darmstadt, Sean Sylvia, Joshua V. Garn, Sarah A. Friedman, Ann M. Weber

**Affiliations:** 1 Department of Epidemiology, Biostatistics and Environmental Health, School of Public Health, University of Nevada, Reno, Reno, NV, United States; 2 Stanford Center on China’s Economy and Institutions, Stanford University, Stanford, CA, United States; 3 Stanford Center for Innovation in Global Health, Stanford University, Stanford, CA, United States; 4 Department of Pediatrics, Stanford University School of Medicine, Stanford, CA, United States; 5 Carolina Population Center, University of North Carolina at Chapel Hill, Chapel Hill, NC, United States; 6 Department of Health Behavior, Policy, and Administration Sciences, School of Public Health, University of Nevada, Reno, Reno, NV, United States

**Keywords:** breastfeeding, breastfeeding promotion, breastfeeding trends, China, cluster analysis, exclusive breastfeeding, rural

## Abstract

**Introduction:**

Longitudinal trends in breastfeeding (BF) are often overlooked in favor of binary or time-to-cessation measures. Characterizing these trends can inform promotion of sustained BF practices. We identified distinct BF profiles among participants of a maternal and child health program.

**Methods:**

The Healthy Future program consisted of community health workers delivering a BF curriculum to mothers through monthly home visits. The program was evaluated in rural Sichuan, China with a cluster-randomized controlled trial (assigned to program versus not). We clustered 6-month postpartum trends (*n* = 949) of maternal-reported infant feeding using dynamic time warping. For each month, participants were categorized as either exclusive breastfeeding (EBF), mixed feeding (MF, feeding breastmilk plus other foods or liquids), or not breastfeeding (NBF). After identifying clusters, we regressed BF profiles on intervention assignment using adjusted multinomial logistic regression.

**Results:**

Cluster analysis revealed seven profiles: always EBF, always MF, never breastfed, EBF until the 5th month, MF until the 5th month, mostly EBF, and NBF from the 3rd month. The intervention was associated with improved odds of always EBF (ROR = 2.61, 95% CI 1.25, 5.42), MF until the 5th month (ROR = 2.52, 95% CI 1.18, 5.39), and NBF from the 3rd month (ROR = 2.82, 95% CI 1.16, 6.87) compared to being never breastfed. Mothers in the never breastfed cluster had the lowest age, education, BF knowledge and attitudes, and decision-making power.

**Discussion:**

Cluster analyses found the intervention significantly improved EBF, particularly in mothers characterized by higher baseline educational attainment and BF knowledge. Targeted efforts are needed to help mothers initiate EBF from birth and continue EBF through month 6.

## Introduction

Benefits of breastfeeding (BF) for child health are well-established, including infection prevention, protection against chronic disease, lower rates of obesity, and enhanced cognitive development (1). For lactating mothers, BF can provide short-term reduction in postpartum depression, stress, anxiety, bleeding, and infection (2). The World Health Organization (WHO) and United Nations International Children’s Emergency Fund (UNICEF) recommend exclusive breastfeeding (EBF) for the first 6 months, aiming to achieve a global EBF prevalence of 70% by 2030 (3). Yet, fewer than half (48%) of infants 0–5 months of age worldwide are currently estimated to be exclusively breastfed (4). In China, the second most populous country in the world, this number drops to 35% despite long-standing national policies and programs for increasing EBF prevalence, including an annual breastfeeding promotion day, implementation of Baby Friendly Hospital Initiatives, and efforts to improve policies for working mothers (5).

The Healthy Future (HF) program was a large-scale trial of a digitally enabled, stage-based curriculum for maternal and child health delivered in households by community health workers (CHWs) in rural, poor China (6). The program sought to increase EBF prevalence as one of its primary outcomes, motivated, in part, by alarmingly poor BF practices and widespread formula use in rural counties of China (7). Intent-to-treat analyses of the trial showed significant improvements among program participants in early initiation of BF and EBF during the first month, but not in 6-month EBF (8).

While the use of a 6-month EBF indicator, defined as the infant was fed exclusively breast milk during the previous day, is accepted practice in program evaluation, it fails to capture dynamic individual circumstances that influence feeding practices over time, effectively discounting time-varying contextual drivers of breastfeeding trajectories. Challenges to EBF that are often beyond mothers’ control (and outside the scope of the intervention) include hospital delivery practices, birth complications and maternal perinatal health, certain child characteristics, and lack of accommodation for BF when returning to work (9–13). Further, new mothers may experience periods of elevated depression and stress, lack of sleep, and increased risk of illness during the first 6 months postpartum (14), possibly thwarting their best intentions to practice EBF at all times. Consequently, interpretations of the success of infant feeding programs that rely on 6-month indicators of feeding practices potentially overlook opportunities in future program design to support sustained breastfeeding in diverse real-world conditions.

To more fully evaluate the impact of the HF intervention on BF practices, we identified six-month BF trend profiles using a time-series cluster analysis, described baseline characteristics of participants in each profile, and estimated the intervention effect on resulting BF trends. Additionally, we estimated the effect of the HF program on time-to-cessation of EBF or any breastfeeding (ABF) for comparison.

## Methods

### Healthy future program

The evaluation of the Healthy Future program enrolled 1,308 pregnant individuals and caregivers with infants under 6 months (8). Participants were selected from 119 townships in four nationally designated poverty counties in rural Sichuan, China. Participants were surveyed at baseline (mid-2021) and 12 months later at endline (mid-2022). The final sample included data for 1,149 caregiver-infant dyads. A detailed description of the study procedures and results are described elsewhere (6, 8). The study received Institutional Research Board approval from Sichuan University (Protocol K2019046), Stanford University (Protocol 44312) and the University of Nevada, Reno (Project 1737966–1).

The study randomly assigned 10 townships per county (40 townships total) to the home-visiting program. Intervention townships were stratified into two delivery arms: (1) the program was delivered to mothers, and (2) the program was delivered to mothers and secondary caregivers (e.g., grandmothers) were encouraged but not required to join. The two intervention delivery arms were combined for our analyses to increase statistical power as the field implementation was similar. Comparative analysis of these two delivery strategies will be reported elsewhere.

The full details of the Healthy Future curriculum are described in the protocol paper (6). In brief, CHWs delivered a digitally enabled, stage-based curriculum during weekly (first month postpartum) and monthly home visits. The BF curriculum included educating mothers on the health benefits of breast milk and colostrum, demonstrating techniques to overcome BF difficulties, and educating about when to introduce complementary foods.

### Outcomes

At endline, participants were asked to report infant feeding practices in the previous 24 h and recall infant feeding behaviors for each month of the first 6 months after birth. Practices included feeding infants breast milk, other liquids (e.g., animal milk, water, formula), semi-solids, and solid foods for each month. We compared monthly retrospective feeding data obtained at endline to 24-h dietary recall data obtained at baseline among the subset of participants with infants at baseline. The correlation between the two measures was 0.76 and did not differ significantly by intervention group. To minimize bias from missing monthly data when using 24-h recall at endline only, we combined monthly and 24-h endline feeding data in our analyses into a single measure.

For the cluster analysis, we calculated a three-category feeding status variable for each month. Infants not fed breast milk for a given month were categorized as not breastfeeding (NBF = 1). Those fed breast milk along with other foods/liquids were labeled mixed feeding (MF = 2). Infants fed only breast milk were exclusive breastfeeding (EBF = 3).

For the time-to-event analysis, we calculated time-to-cessation of EBF and time-to-cessation of ABF in months using retrospective feeding data. We considered the time the baby was brought home after birth as baseline. If a child continued EBF or ABF through the first 6 months or, if they were below 6 months old, until their current age at the time of the endline survey, they were right censored, for the given outcome.

### Covariates

Based on the literature (15), covariates included for all adjusted regressions were infant (age, sex, birth through c-section, birthweight, gestational age, and born at baseline), parent (high school education, age, parity), and household (household wealth index, mother previously migrated, presence of household members, household located within town, county) baseline characteristics. We also included maternal sources of caregiving knowledge (i.e., hospital, internet), BF attitudes, BF knowledge, number of times hands were washed the prior day, mental health, perceived decision-making power, decision-making conflict, and social support.

Maternal social support was measured using the Multidimensional Scale of Perceived Social Support (MSPSS) (16). The Iowa Infant Feeding Attitude Scale (IIFAS) was used to measure BF attitudes (17). Maternal BF knowledge was measured using 12 questions on infant and maternal diet (Supplementary Table S1). Higher scores corresponded with higher support, attitudes, and knowledge, respectively. Mental health was scored using the Depression Anxiety Stress Scale-21 (DASS-21), with higher scores reflecting more symptoms of poor mental health (18). Mothers were scored on household decision responsibilities in 10 topics (e.g., spending money, choosing to breastfeed), with higher scores indicating greater maternal decision-making power (Supplementary Table S2) (19, 20). Mothers were asked about spousal conflict (e.g., conflict on BF), with a higher number indicating more conflict.

### Statistical methods

We plotted the average prevalence of infant feeding behaviors (NBF, MF, EBF) for each of the first 6 months postpartum by Healthy Future program assignment.

We conducted a time-series cluster analysis to understand the profiles of BF behavior across the first 6 months postpartum. Participants were grouped based on their BF trends using the dynamic time warping (DTW) package in R (21), which measured how similar BF trends for each participant were to another using a DTW distance score. Participants with similar trends received low distance scores and were grouped together. The method requires pre-specification of a maximum number of clusters into which participants can be grouped, which was selected by optimizing the scores of various indices (i.e., silhouette, score function, Calinski-Harabasz, Davies-Bouldin, modified Davies-Bouldin, Dunn, and COP indices). We estimated cluster results for 14 different models where the maximum cluster number was set from 2 to 15. The scores of the seven indices were compared to identify the maximum cluster number with the best fit (i.e., varied by index). We inspected the clustered trends of the model with the chosen maximum cluster number and further combined similar clusters together based on visual and conceptual similarities in trends. Baseline characteristics of participants of the predominant cluster groups were tabulated. Finally, we used a multinomial logistic regression model to estimate program effects on the final set of BF trends, adjusting for baseline characteristics and clustering of the intervention within townships. As a sensitivity analysis, we compared the results generated by the DTW quantitative clustering method to those from a qualitative approach which used pre-defined cluster rules (Supplementary Table S3).

Separate from the cluster analysis, we conducted time-to-cessation of EBF and time-to-cessation of ABF analyses and plotted Kaplan–Meier time-to-cessation curves by intervention arm. We used inverse probability weighted (IPW) Weibull models to estimate the average treatment effect (ATE) on number of months until EBF and ABF cessation. Unlike more commonly used Cox proportional-hazards model, Weibull models do not require hazards to be proportional. Additionally, using inverse treatment weights allowed us to estimate potential outcomes and, by extension, the ATE. We modeled the probability of being treated by regressing (i.e., using logistic regression) treatment on the baseline covariates mentioned previously. The inverse of these weights was used in the Weibull model to estimate the potential outcome means under treatment and control for all participants. The model estimated the mean number of months until cessation of EBF and ABF, conditional on everyone having been treated, and the mean conditional on no one having been treated. The difference of these means was the ATE of the Healthy Future intervention on EBF and ABF.

As a sensitivity analysis, we used the Cox proportional-hazards model to estimate the program effect on the hazard of stopping EBF and ABF, adjusted for baseline covariates. As infants born through c-section, with low birthweight, or preterm have a higher chance of being fed formula, the model adjusted for varying baseline hazards by birth characteristics (15).

## Results

### Analytic sample

Some participants (*n* = 145) were not asked about feeding outcomes due to logistical challenges during endline data collection during the COVID-19 pandemic. These participants were excluded from all analyses. Missing baseline characteristics were imputed using the means (for continuous variables) or modes (for binary variables) of the characteristics for participants within the same township (Supplementary Table S4). Our analytic sample included 1,004 participants. Our DTW cluster analysis required data for all months, for all participants. However, some infants (*n* = 123) were younger than 5 months at endline, and 68 had missing feeding data only for month 6. For these 68 individuals, month 6 feeding data were imputed using a multinomial logistic regression model with program status, feeding outcomes for months one to five, and baseline characteristics. Before imputation, the correlation between feeding data from months five and six was 0.917. After imputation, this correlation remained high at 0.910. The remaining infants (*n* = 55) with missing feeding data from earlier months were dropped, leaving a final sample of 949 participants (control *n* = 550, intervention *n* = 399) for our cluster analysis.

### Average feeding behaviors in the first 6 months

**Figure 1:**
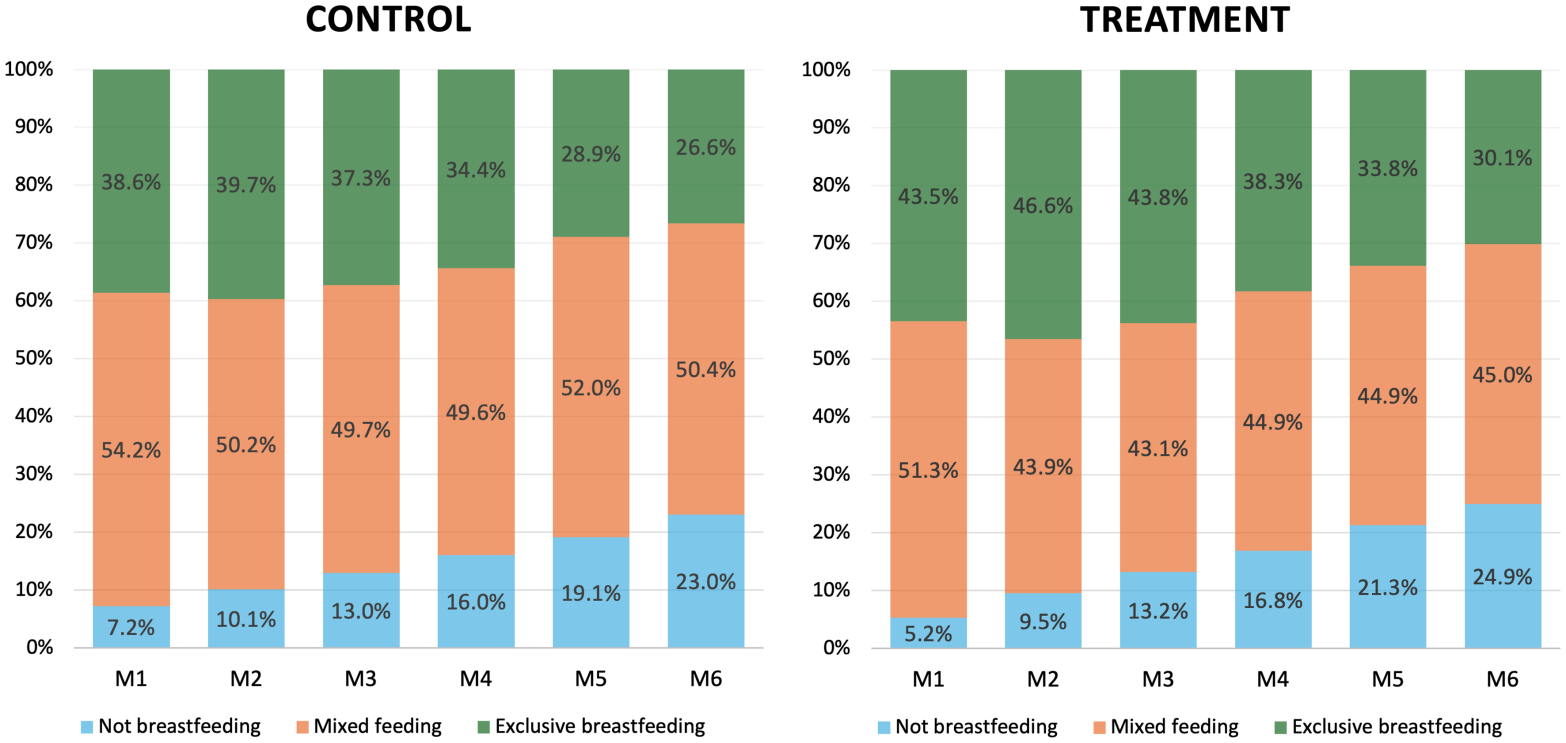
Average infant feeding behaviors during the first 6 months postpartum shown by intervention assignment, among participants of the Healthy Future study (*n* = 1,003). Feeding behaviors were grouped into three categories: Not breastfeeding (i.e., not fed breast milk), mixed feed (i.e., fed breast milk + something else), and exclusive breastfeeding (i.e., fed only breast milk).

EBF was practiced by a minority of participants across all months and intervention groups (Figure 1). EBF prevalence remained stable for the first 2 months in the control group (Month 1 = 38.6%, Month 2 = 39.7%) but increased from 43.5 to 46.6% from Month 1 to 2 among program participants. The increase in EBF in the second month in the intervention group was accompanied by a decrease in MF prevalence (Month 1 = 51.3%, Month 2 = 43.9%). EBF declined over Months 2–6 in both groups; across all months, EBF prevalence was higher in the intervention compared to the control group, reaching 30.1% in the intervention group and 26.6% in the control group during Month 6.

### Cluster analysis

**Figure 2:**
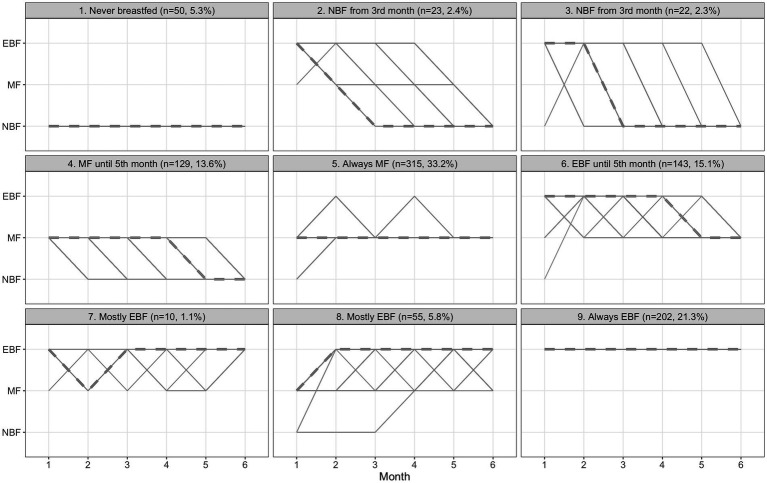
Dynamic time warping clustered trends in infant feeding behaviors during the first 6 months postpartum among participants of the Healthy Future study (*n* = 949). Solid lines represent individual trends within a cluster and the dashed lines represent the typical trend within a cluster. NBF = Never breastfed, MF = mixed feeding (i.e., fed breast milk + something else), and EBF = exclusive breastfeeding (i.e., fed only breast milk). Clusters 2 and 3 were combined into a single cluster, “NBF from 3rd month.” Clusters 7 and 8 were combined into a single cluster, “Mostly EBF”.

Participants were grouped into nine clusters (Figure 2), which we further classified into seven profiles by combining small clusters (*n* < 30) with similar trends. Three of the seven profiles showed consistent feeding status across all months; these were labeled as always EBF (*n* = 202, 21.3%), always MF (*n* = 315, 33.2%), and never breastfed (*n* = 50, 5.3%). Two profiles showed consistent feedings until the 5th month: EBF until 5th month (*n* = 143, 15.1%) and MF until 5th month (*n* = 129, 13.6%). The last two profiles showed trends of mostly EBF (*n* = 65, 6.9%) and NBF from the 3rd month (*n* = 45, 4.7%).

Mothers who never breastfed were on average younger (58.0%), less educated (18.0% completed high school), had lower BF knowledge (16.0%), and had lower decision-making power (32.0%) compared to other groups (Table 1). Additionally, fewer had a secondary caregiver in the household (60.0%), had previously migrated for work (66.0%), and lived in households with high household assets (36.0%). Mothers in the never breastfed, NBF from 3rd month, and mostly EBF clusters had more negative BF attitudes (prevalence of mothers with high BF attitudes <37.0%) compared to other groups (Table 1). Mostly EBF mothers had high decision-making power (52.3%). Mothers in the always EBF cluster had the highest levels of BF knowledge (53.0%). Other clusters had moderate knowledge levels, i.e., between 32.7 and 38.5%. Mothers consistent in their feeding behaviors (i.e., never breastfed, always MF, or always EBF) had better mental health (NBF = 48.0%, MF = 48.3%, EBF = 49.5%) than those who were not consistent.

**Table 1:** Baseline characteristics of Healthy Future participants by infant feeding trend clusters.

Baseline characteristics (%)	Never breastfed (*n* = 50)	NBF from 3rd month (*n* = 45)	MBF to 4th month (*n* = 129)	Always MBF (*n* = 315)	EBF to 4th month (*n* = 143)	Mostly EBF (*n* = 65)	Always EBF (*n* = 202)
Infant							
In intervention group	36.0%	48.9%	46.5%	38.7%	42.0%	32.3%	47.5%
Born at baseline	80.0%	73.3%	63.6%	66.7%	53.8%	41.5%	72.3%
Delivered by C-section	58.0%	55.6%	65.1%	57.1%	55.9%	49.2%	51.5%
Preterm birth (<37 weeks)	8.0%	4.4%	6.2%	4.1%	6.3%	1.5%	4.5%
Female sex	60.0%	53.3%	50.4%	47.9%	51.0%	52.3%	52.5%
Low birth weight (<2.5 kg)	8.0%	6.7%	7.8%	4.4%	2.8%	1.5%	5.9%
Parent							
Younger mother (age<median)	58.0%	48.9%	48.1%	39.0%	43.4%	46.2%	44.1%
Younger father (age<median)	42.0%	31.1%	45.7%	45.7%	55.2%	49.2%	51.5%
At least 1 prior birth	66.0%	66.7%	69.8%	65.1%	75.5%	64.6%	67.8%
Mother completed high school	18.0%	33.3%	41.1%	36.8%	39.9%	47.7%	39.6%
Father completed high school	32.0%	40.0%	36.4%	42.5%	42.7%	46.2%	48.0%
Mother							
High breastfeeding attitudes (score>median)	36.0%	35.6%	49.6%	46.0%	46.9%	36.9%	52.0%
High breastfeeding knowledge (score>median)	16.0%	35.6%	34.1%	32.7%	38.5%	36.9%	53.0%
High handwashing behavior (score>median)	54.0%	37.8%	44.2%	44.8%	39.9%	46.2%	46.0%
Primary caregiving info source is hospital	60.0%	53.3%	44.2%	51.1%	54.5%	46.2%	50.0%
Primary caregiving info source is internet	10.0%	8.9%	10.9%	13.7%	15.4%	18.5%	14.9%
Good mental health (score<median)	48.0%	40.0%	38.0%	48.3%	42.0%	40.0%	49.5%
High decision-making power (score>median)	32.0%	42.2%	31.8%	40.6%	35.0%	52.3%	40.6%
Low household conflict (score = 0)	64.0%	66.7%	58.1%	64.4%	64.3%	63.1%	65.3%
High social support (score>median)	46.0%	44.4%	46.5%	47.6%	52.4%	44.6%	51.0%
Household							
Father present at baseline	92.0%	93.3%	96.9%	94.3%	95.8%	95.4%	93.1%
Grandparents present at baseline	88.0%	88.9%	86.8%	79.4%	84.6%	83.1%	82.2%
Secondary caregiver present at baseline	60.0%	71.1%	74.4%	75.9%	79.0%	76.9%	73.3%
High household assets (index>median)	36.0%	44.4%	55.0%	50.5%	53.1%	44.6%	59.4%
Mother previously migrated for work	66.0%	91.1%	83.7%	85.4%	86.0%	89.2%	84.7%
House is located within the town	46.0%	42.2%	45.7%	44.1%	47.6%	44.6%	51.0%

Color Scale:

**Table:** 

0–9.9%	10–19.9%	20–29.9%	30–39.9%	40–49.9%	50–59.9%	60–69.9%	70–79.9%	80–89.9%	90–100%

**Figure 3:**
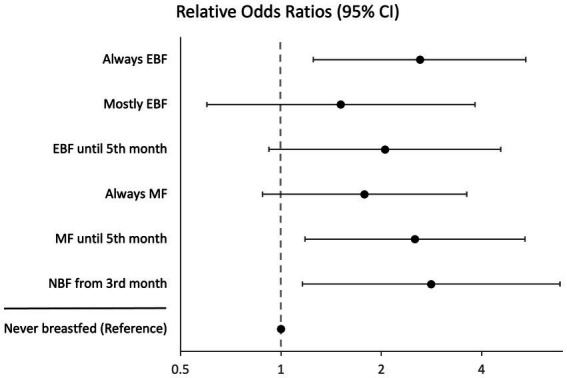
Results of multinomial logistic regression of dynamic time warping clustered infant feeding trends on Healthy Future program. The model was adjusted for baseline characteristics, and the standard errors were adjusted for clustering by township. NBF, Not Breastfeeding; MF, mixed feeding; EBF, exclusive breastfeeding.

Supplementary Figure S1 compares the prevalence of BF trends between intervention and control groups. Prevalence of always MF mothers (Control = 35.1%, Intervention = 30.6%) in the intervention group decreased, while always EBF (Control = 19.3%, Intervention = 24.1%) increased. Multinominal regression of clustered trends on intervention showed the intervention was associated with higher odds, relative to the control, of always EBF (Relative odds ratio (ROR) = 2.61, 95% CI 1.25, 5.42), MF until the 4th month (ROR = 2.52, 95% CI 1.18, 5.39), and NBF from the 3rd month (ROR = 2.82, 95% CI 1.16, 6.87) with reference to never breastfed (Figure 3).

The qualitative clustering analysis yielded 10 BF profiles: always EBF (*n* = 202), always MF (*n* = 305), never breastfed (*n* = 50), early cessation of EBF (*n* = 170), early cessation of ABF (*n* = 129), mostly EBF (*n* = 50), mostly MF (*n* = 21), mostly NBF (*n* = 1), equal EBF and MF (*n* = 17), and unclassified (*n* = 4) (Supplementary Table S5). Supplementary Figure S2 compares the participant membership between DTW-generated and qualitative BF profiles, indicating several similarities in membership. Furthermore, multinomial regression of qualitatively-generated profiles showed similar results compared to the DTW-profile regression. The intervention was associated with higher odds, relative to the control, of always EBF (ROR = 2.64, 95% CI 1.27, 5.48), early cessation of ABF (ROR = 2.54, 95% CI 1.19, 5.44), and early cessation of EBF (ROR = 2.26, 95% CI 1.03, 4.93) with reference to never breastfed (Supplementary Figure S3).

### Time-to-event analysis

Time-to-event curves of EBF show similar trends in both treatment and control groups (Supplementary Figure S4). Time-to-event curves of ABF show similar trends in time-to-cessation between the intervention and control groups. Inverse probability of treatment weighted Weibull models did not provide evidence of a link between the intervention and time-to-cessation of EBF (Difference in mean months to cessation = −0.02, 95% CI −0.36, 0.32) or ABF (Difference in mean months to cessation = −0.01, 95% CI −0.38, 0.36) (Table 2). Results of the adjusted Cox proportional hazards models (EBF hazard ratio = 0.33, 95% CI 0.11, 1.03; ABF hazard ratio = 1.21, 95% CI 0.81, 1.80) matched those of the Weibull models.

**Table 2:** Results of Weibull and Cox proportional hazards models estimating the effect of the Healthy Future intervention on time-to-cessation of exclusive breastfeeding (EBF) and any breastfeeding (ABF) during the first 6 months postpartum.

Models	EBF (*n* = 408)	ABF (*n* = 940)
Inverse probability of treatment weighted Weibull model^b^		
Mean difference in number of months until cessation (95% CI^a^)	−0.02 (−0.36, 0.32)	−0.01 (−0.38, 0.36)
Mean number of months until cessation had everyone been assigned to the control (95% CI^a^)	2.96 (2.70, 3.21)	3.31 (3.04, 3.57)
Cox proportional hazards model^c^		
Unadjusted hazard ratio (95% CI^a^)	0.76 (0.52, 1.11)	0.99 (0.71, 1.38)
Adjusted^d^ hazard ratio (95% CI^a^)	0.33 (0.11, 1.03)	1.21 (0.81, 1.80)

^a^All confidence intervals were adjusted for clustering by township using robust standard errors. ^b^The Inverse probability of treatment weighted Weibull models were adjusted for baseline characteristics. ^c^All Cox proportional hazards models accounted for different baseline hazards by gestational age, birth weight, and birth method. ^d^Adjusted Cox models included baseline characteristics.

## Discussion

### Intervention effects

The prevalence of EBF increased in program mothers from month one to two (Figure 1), suggesting the intervention improved initiation and early cessation of EBF. The cluster analyses offer supporting evidence, indicating that the intervention improved odds of BF at the first month, including EBF, compared to never BF. The Healthy Future program included components to encourage mothers to breastfeed by promoting the benefits of colostrum and breastmilk. Additionally, CHWs worked with mothers on BF techniques to overcome barriers and complications that may discourage mothers from continuing BF. The intervention also improved the odds of mostly EBF compared to never BF, indicating the intervention may have helped some mothers continue EBF, albeit with intermittent challenges.

Though cluster analyses found evidence of intervention effects, time-to-event analyses did not. This could be because time-to-event analyses penalized mothers in the intervention group who did not breastfeed or exclusively breastfeed during the first month but worked with the CHWs to initiate BF or EBF in the second month. The program may have helped women to overcome challenges that initially interfered with EBF, but this success was obscured with time-to-cessation measures of BF. Another possible reason for the varying results is a difference in the analytical sample. The cluster analyses included more individuals (*n* = 949) than the time-to-event analysis on EBF (*n* = 408), which excluded mothers who did not exclusively breastfeed during the first month postpartum.

Future evaluations should consider the overall trend of BF instead of binary indicators averaged for the full 6 months’ duration and time-to-cessation measures. By examining 6-month feeding profiles, study findings have the potential to inform the design of more precisely targeted BF support strategies that align delivered content with evolving needs and circumstances of different caregivers. Such precision targeting can be operationalized through a digitally enabled, stage-based curriculum, such as the one developed for the Healthy Future program, so that content is responsive to families’ breastfeeding intentions and challenges encountered in realizing them.

### Factors associated with BF profiles

Mothers who never breastfed during the 6-month period had the lowest age, education, BF knowledge, BF attitudes, and decision-making power of any cluster, and these characteristics match those of mothers who did not breastfeed during the first month. These findings are consistent with what is well known in the field: caregiving experience, feeding and general knowledge, and BF attitudes are strongly tied to EBF initiation and continuation (15). Preterm birth and low birth weight were also found to be associated with NBF, consistent with prior research (22). Similarly, mothers who mostly (but not always) exclusively breastfed had low baseline BF attitudes and BF knowledge, but differed by having high decision-making power. One explanation might be that their high decision-making power allowed them to initiate EBF, but their lack of BF knowledge and low attitudes toward BF hindered their willingness to consistently exclusively breastfeed. Particularly, high baseline BF knowledge was strongly associated with initiation of EBF and continuation of EBF for 6 months compared to MF or NBF.

Mental health may be associated with feeding behavior over time. Mothers with consistent feeding profiles (e.g., always MF, EBF, NBF) had better mental health than those with inconsistent feeding profiles (e.g., mostly EBF). Postpartum depression and stress are inversely associated with BF and are known to fluctuate during the postpartum period (23). Months of high stress or depression could hinder continuation of intended feeding behaviors. Further research into these patterns and influences on BF is warranted.

### Months 1 and 5

Months 1 and 5 were key in determining BF profile membership. The most prevalent profiles were always MF, always EBF, EBF until the 5th month, and MF until the 5th month. Mothers who practiced EBF or MF in the first month tended to continue these behaviors up to 5 or 6 months, consistent with prior research (24). In rural China, the prevalence of early initiation of BF (22.2%) in the hospital is low, and supplementation with formula (77.6%) and water (61.8%) is high (7). Additionally, gifts of formula are commonly provided by hospitals to mothers (25). Hospital supplementation and formula marketing were shown to decrease rates of EBF in China (26). Globally, formula marketing has become more prevalent and has contributed to decreased national breastfeeding rates, particularly in upper-middle- and high-income countries (27). Within China, the Healthy Future intervention may have helped to improve rates of BF initiation by collaborating with mothers, their families, and hospital staff during antenatal care and childbirth, and closely engaging with families through the first month postpartum. Addressing mothers’ barriers, knowledge, attitudes, and motivation toward BF during the antenatal period and the first month may be crucial to improving continuation of EBF. Incorporating telehealth and mobile-based communication, e.g., WeChat messages, during the first month may help address concerns more quickly and empower mothers compared to home visits alone (28). Furthermore, efforts to regulate formula marketing may contribute to increased EBF (29).

Month five was when mothers who were exclusively breastfeeding started to incorporate complementary foods, whereas mothers who were MF stopped BF and switched entirely to complementary foods. Similar trends have been observed in a cohort of British mothers (30). Studies have shown that during this time, mothers in lower, lower-middle, and upper-middle income countries have numerous rationales for introducing new foods and even full cessation of BF (9–13). Infants are larger at this age and show increased attention toward foods that family members eat (9). Additionally, mothers may want to introduce cultural foods early. Mothers may worry that their infants are not getting enough food through breastmilk alone, especially if they are struggling to breastfeed or with a perception of inadequate breastmilk production (24). Mothers may also have work obligations that force them to stop BF earlier than the recommended 6 months, opting instead for formula (13). Further efforts should be made to understand and empathize with mothers in their struggles and encourage them to delay supplementation until the infant turns 6 months old. Mothers may also benefit from lactation consultation during these months if they are concerned about their breastmilk supply or are struggling with BF. Additionally, stakeholders should collaborate with policy makers to expand workplace policies and protections for lactating mothers, e.g., designated break times, private spaces to breastfeed, anti-discrimination laws.

### Strengths and limitations

To our knowledge, this was the first study to employ a time-series cluster analysis to identify distinct trends in BF during the first 6 months postpartum. The analysis allowed for the characterization of the nuanced real-world BF behaviors of study participants and estimation of positive intervention effects on 6-month feeding behavior profiles that were obscured in more traditional analytic approaches. Additionally, we compared the results of our quantitatively driven DTW clustering method with a qualitative approach and found similar results, providing evidence to support the stability of our identified clusters.

A limitation of the study is potential recall issues of monthly feeding data. Mothers of older infants may have worse recall of feeding behaviors than mothers of younger infants. However, high correlation was found between the monthly recall measures utilized in the cluster analysis and standard 24-h recall responses. Moreover, this correlation did not vary by intervention arm, suggesting that possible recall bias was not differential. Future studies will incorporate longitudinal monthly measurement of feeding practices. Despite random assignment, the program effect estimates may be biased from attrition and loss of balance across treatment arms. Though adjustments were made for numerous infant, parent, maternal, and household baseline characteristics associated with BF, some residual bias may have remained. Additionally, statistically insignificant findings may be due to low sample size rather than true null effects. Our results are limited to the cultural and regional context of rural China and may not be representative of rural areas in other countries - further research in other settings is needed.

## Conclusion

We demonstrated the power of longitudinal cluster analysis to move from “if” breastfeeding stops to “how” it changes over time, providing a more actionable evidence base for targeted interventions. The identification of the “never breastfed” cluster, composed of mothers with the lowest socioeconomic and empowerment indicators, highlights a vulnerable subgroup requiring intensive, multifaceted support from birth. This speaks directly to issues of health equity. The lessons learned from characterizing these complex feeding patterns and the differential impact of an intervention are applicable beyond rural China, informing public health strategies in diverse settings aiming to improve maternal and child nutrition.

## Data Availability

The raw data supporting the conclusions of this article will be made available by the authors, without undue reservation.
