# Supplementary material for: Uncovering hidden patterns: use of infant feeding profiles in the first 6 months postpartum to inform the effectiveness of breastfeeding promotion programs
Source: Front Public Health. 2026 Apr 17;14:1754431. doi: 10.3389/fpubh.2026.1754431 (PMC13132859; doi:10.3389/fpubh.2026.1754431)
Supplement: Supplementary file 1 [file Supplementary_file_1.docx]

**Uncovering Hidden Patterns: Use of infant feeding profiles in the first 6 months postpartum to inform the effectiveness of breastfeeding promotion programmes**

Siva Balakrishnan^1^, PhD; Yunwei Chen^2,3^, PhD; Gary L. Darmstadt^2,3,4^, MD; Sean Sylvia^5^, PhD; Joshua V. Garn^1^, PhD; Sarah A. Friedman^6^, PhD; and Ann M. Weber^1^, PhD.

1. Department of Epidemiology, Biostatistics and Environmental Health, School of Public Health, University of Nevada, Reno, Reno, NV, 89557, USA
2. Stanford Center on China’s Economy and Institutions, Stanford University, Stanford, CA, 94305, USA
3. Stanford Center for Innovation in Global Health, Stanford University, Stanford, CA, 94305, USA
4. Department of Pediatrics, Stanford University School of Medicine, Stanford, CA, 94305, USA
5. Carolina Population Center, University of North Carolina at Chapel Hill, Chapel Hill, NC, 27516, USA
6. Department of Department of Health Behaviour, Policy, and Administration Sciences School of Public Health, University of Nevada, Reno, Reno, NV, 89557, USA

**Corresponding author:** Siva Balakrishnan, PhD. Email: [balakrs@stanford.edu](mailto:balakrs@stanford.edu)

Figure S1. Clustered infant feeding trends by Healthy Future programme status among participants of the Healthy Future study (n=949). NBF = Not breastfeeding, MF = mixed feeding (i.e., fed breast milk + something else), and EBF = exclusive breastfeeding (i.e., fed only breast milk).

Figure S2. Comparison between dynamic time warping (DTW)-generated and qualitatively-generated breastfeeding clusters. NBF = not breastfeeding, ABF = any breastfeeding, MF = mixed feeding, and EBF = exclusive breastfeeding.


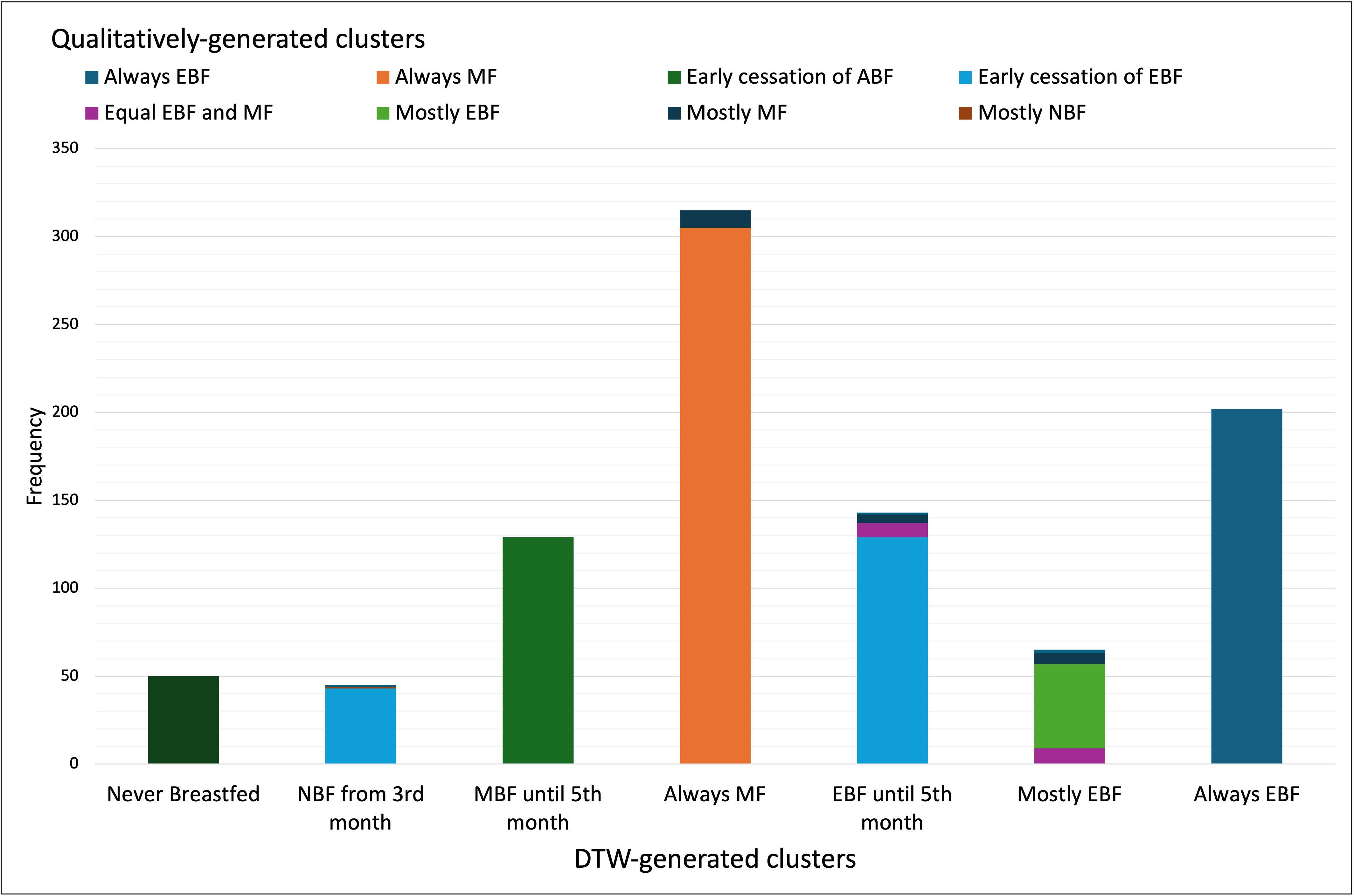


Figure S3. Results of multinomial logistic regression of qualitatively-generated breastfeeding profiles on Healthy Future programme assignment. The model was adjusted for baseline characteristics, and the standard errors were adjusted for clustering by township. ABF = any breastfeeding, MF = mixed feeding, and EBF = exclusive breastfeeding.


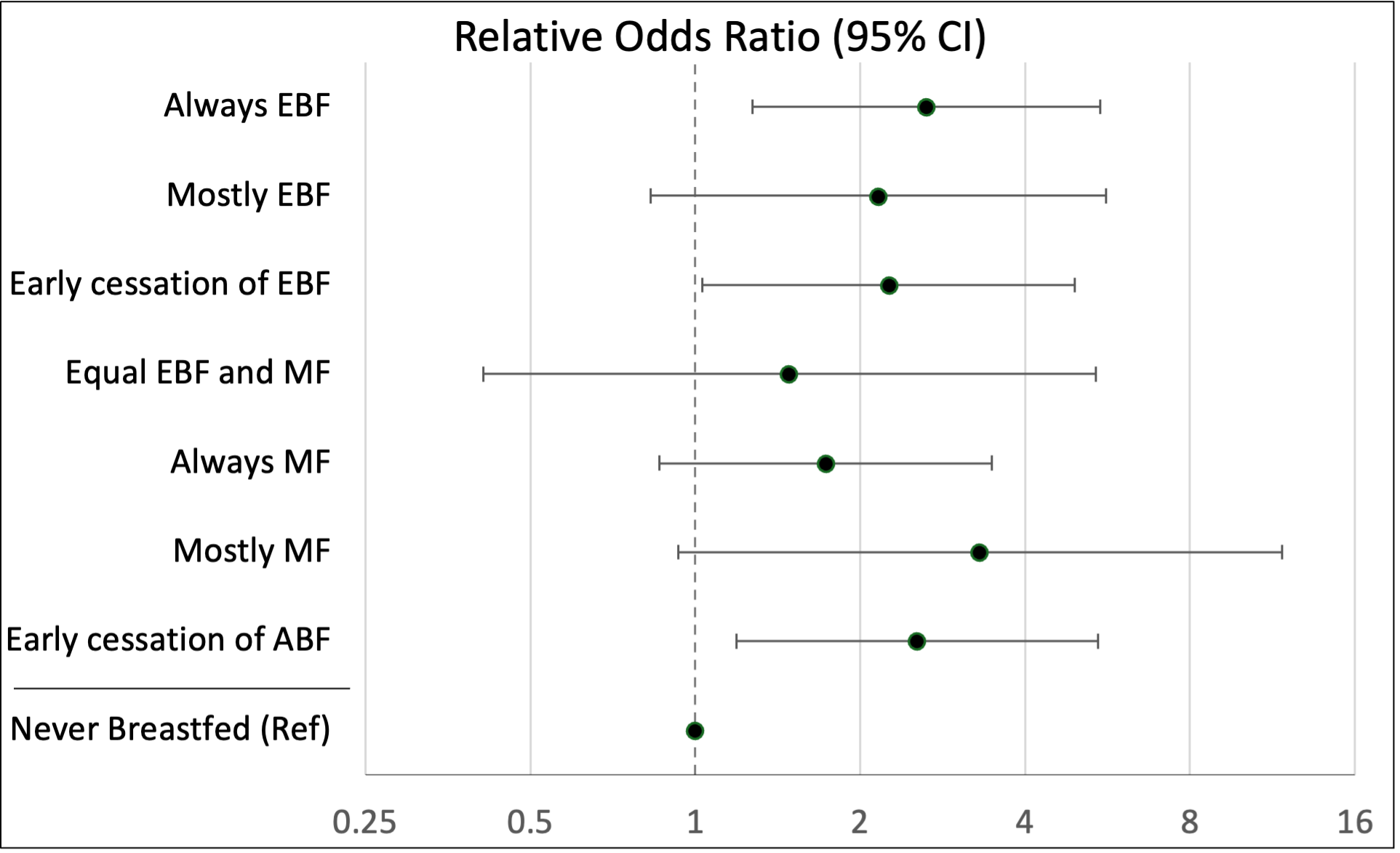


Figure S4. Kaplan-Meier time-to-event curves of time-to-cessation of exclusive breastfeeding (EBF) and any breastfeeding (ABF) by Healthy Future programme status.

Table S1. Survey instrument measuring infant and caregiving knowledge. Correct answers were summed to get the final score, which could range between 0 (low knowledge) and 14 (high knowledge).

| **Baseline caregiving and feeding knowledge score (0-14)** | **Correct** |
| --- | --- |
| 1. The first milk after birth is bad for your baby and should be discarded. Do you think it’s right? |  |
| 2. Women with small breasts cannot produce enough milk to feed a baby. Do you think it’s right? |  |
| 3. It is important to feed your baby water in addition to breastmilk. Do you think it’s right? |  |
| 4. Breastfeeding mothers should drink more than usual in order to ensure a good milk supply |  |
| 5. When should you breastfeed your child? |  |
| 6. When should you start to introduce water to your child? |  |
| 7. When should you start to introduce formula to your child? |  |
| 8. When should you start to introduce soft or semi-solid foods to your child? |  |
| 9. Which of the following foods is the best source of iron? |  |
| 10. What is the best way to know if your child has anemia? |  |
| 11. If you are feeling sad or overwhelmed, which of the following is a good strategy for coping? |  |
| 12. What is the best way to prevent your baby from getting a cold? |  |
| 13. Do you think babies who are not breastfed get sick easier, compared to the babies who are breastfed? |  |
| 14. If the baby has poor immunity, how do you think it affects the baby’s growth? |  |
| **SUM** |  |

Table S2. Survey instrument measuring both maternal household decision-making power and conflict. Participants were asked about decision-making responsibilities and conflicts on ten topics. Responses were scored (see table) and summed to get the final score. Decision-making power scores ranged from 10 (low power) to 30 (high power). Decision-making conflict scores ranged from 0 (no conflict) to 10 (high conflict).

| **Baseline decision-making power and conflict scores** |
| --- |
| 1. Decision-making power: Who in your family usually has the final say on the decision of … ? 2. Respondent (Score=3) 3. Jointly with others in household (Score=2) 4. Others in the household (Score=1) |
| 1. Decision-making conflict: In the last month, has there been a disagreement about this type of decision? (final score ranges 0-10) 2. Yes (Score=1) 3. No (Score=0) 4. This decision has not come up in the last month (Score=0) |
| **Topics**  1. What food to buy for family meals?  2. Whether to purchase small household items such as an utensils, lamp?  3. Whether or not you should work to earn money?  4. Whether to purchase major goods for the household such as a TV, furniture?  5. How the household earnings are spent?  6. Obtaining health care for yourself?  7. Exclusively breastfeed newborn for 6 months?  8. What foods to feed the child?  9. What to do if the child falls sick?  10. How much to spent on health care for the child? |

Table S3. Rules for qualitatively clustering breastfeeding trends.

| **Rules for manual clustering of BF trends** |
| --- |
| 1. Participants with consistent feeding behavior across the six months were marked as ALWAYS EBF (n=202), ALWAYS MF (n=305), or NEVER BREASTFED (n=50). |
| 2. Participants who exclusively breastfed for the first 'X' number of months and then dropped to mixed feeding or not breastfeeding for the remaining months were labelled as EARLY CESSATION OF EBF (n=170). Participants who were mixed breastfeeding for the first 'X' number of months and dropped to not breastfeeding for the remaining months were labelled as EARLY CESSATION OF ABF (n=129). |
| 3. Participants with 4 or 5 months of EBF were labelled MOSTLY EBF (n=50). Participants with 4 or 5 months of MF were labelled MOSTLY MF (n=21). Participants with 4 or 5 months of NBF were labelled MOSTLY NBF (n=1). |
| 4. Participants with 3 months of EBF and 3 months of MF were classified and EQUAL EBF AND MF (n=17). |
| 5. The remaining participants were labelled UNCLASSIFIED (n=4). |

Table S4. Number of missing observations in each baseline characteristic.

|  |  | **Number of missing** |
| --- | --- | --- |
| **Child** | Birthweight | 9 |
|  | C-section | 3 |
|  | Endline age | 1 |
|  | Gestational age | 11 |
|  | Sex | 1 |
| **Maternal** | Age | 7 |
|  | Breastfeeding attitudes score | 5 |
|  | Caregiving knowledge score | 17 |
|  | Decision-making conflict score | 16 |
|  | Decision-making power score | 19 |
|  | Education | 10 |
|  | Handwashing score | 14 |
|  | Mental health score | 14 |
|  | Migration status | 2 |
|  | Parity | 32 |
|  | Primary info source is hospital | 14 |
|  | Primary info source is internet | 14 |
|  | Social support score | 14 |
| **Paternal** | Age | 61 |
|  | Education | 71 |
| **Household** | Household asset index | 3 |

Table S5. Breastfeeding profiles produced by the qualitative clustering analysis. ABF = any breastfeeding, MF = mixed feeding, and EBF = exclusive breastfeeding. Individual 6-month postpartum feeding trends: 1=no breastfeeding, 2=mixed feeding, 3=exclusively breastfeeding for that month.

| **Breastfeeding profiles** | **Individual trend** | **N** |
| --- | --- | --- |
| Always EBF (n=202) | 333333 | 202 |
| Always MF (n=305) | 222222 | 305 |
| Never Breastfed (n=50) | 111111 | 50 |
| Early cessation of EBF (n=170) | 333332 | 33 |
|  | 333322 | 32 |
|  | 333222 | 26 |
|  | 332222 | 20 |
|  | 322222 | 18 |
|  | 331111 | 7 |
|  | 333321 | 6 |
|  | 311111 | 5 |
|  | 333111 | 5 |
|  | 321111 | 4 |
|  | 333211 | 4 |
|  | 332111 | 2 |
|  | 332211 | 2 |
|  | 333331 | 2 |
|  | 322111 | 1 |
|  | 322211 | 1 |
|  | 322221 | 1 |
|  | 332221 | 1 |
| Early cessation of ABF (n=129) | 211111 | 36 |
|  | 222111 | 26 |
|  | 222211 | 25 |
|  | 222221 | 23 |
|  | 221111 | 19 |
| Mostly EBF (n=50) | 233333 | 27 |
|  | 233332 | 6 |
|  | 323333 | 4 |
|  | 223333 | 3 |
|  | 133333 | 2 |
|  | 333223 | 2 |
|  | 333311 | 2 |
|  | 233233 | 1 |
|  | 332323 | 1 |
|  | 333233 | 1 |
|  | 333323 | 1 |
| Mostly MF (n=21) | 122222 | 4 |
|  | 232222 | 4 |
|  | 222233 | 3 |
|  | 233222 | 3 |
|  | 222223 | 2 |
|  | 222322 | 2 |
|  | 223322 | 1 |
|  | 322322 | 1 |
|  | 323222 | 1 |
| Mostly NBF (n=1) | 133111 | 1 |
| Equal EBF and MF (n=17) | 233322 | 5 |
|  | 222333 | 4 |
|  | 223332 | 2 |
|  | 323322 | 2 |
|  | 223323 | 1 |
|  | 232332 | 1 |
|  | 233223 | 1 |
|  | 332232 | 1 |
| Unclassified (n=4) | 111233 | 2 |
|  | 133322 | 1 |
|  | 233321 | 1 |
